# Retrospective Analysis of the Pattern and Incidence of Maxillofacial Emergencies: A Hospital-Based Study

**DOI:** 10.7759/cureus.93153

**Published:** 2025-09-24

**Authors:** Neha Bhutiani, Navneet Singh, Harpreet Grewal

**Affiliations:** 1 Orthodontics and Dentofacial Orthopedics, Department of Dentistry, University College of Medical Sciences & Guru Teg Bahadur Hospital, Delhi, IND

**Keywords:** dental emergencies, hard tissue injury, maxillofacial emergencies, orofacial trauma, soft tissue injury, trauma

## Abstract

Introduction

Oral and maxillofacial emergencies are frequently encountered and mandate urgent attention in the emergency departments of any healthcare facility. The tertiary healthcare institutions are often the apex referral centers for such complex and life-threatening medical conditions. Thus, they should be well-equipped to mitigate the morbidity or mortality arising from any emergency medical condition. The current study investigates the incidence and characteristics of cases of oro-maxillofacial emergencies that were reported to the emergency department (ED) of a tertiary care hospital over six months.

Methods

This retrospective study involved an analysis of records of the patients reporting with oro-maxillofacial emergencies to the ED of our hospital over six months (January 1-June 30, 2024). Data regarding age, gender, presenting complaints, etiology, and diagnosis were extracted and anonymized. Cases were classified as traumatic or non-traumatic. Patients with multiple injuries were recorded under the most severe diagnosis. Fractures were categorized using Dingman-Natvig and Le Fort classification systems. Statistical analysis was performed using IBM SPSS Statistics for Windows, Version 25 (Released 2015; IBM Corp., Armonk, New York, United States), applying Chi-square tests and logistic regression to identify associations between risk factors and types of trauma (p<0.05).

Results

A total of 1,117 patients reported to the ED with oro-maxillofacial emergencies during the study period, comprising 841 male patients (75.3%) and 276 female patients (24.7%), with a mean age of 28.75 ± 15.43 years. The majority of patients (n=396, 35.5%) were in the age group of 21-30 years. Of these, 208 patients presented with non-traumatic emergencies, primarily due to pain from impacted wisdom teeth (n=168; 102 male and 66 female patients) and carious teeth (n=40; 22 male and 18 female patients). Out of the total patients (n=1,117), those with traumatic etiology included soft tissue injuries in 506 patients (45.2%), tooth fractures in 136 (12.2%), facial swelling in 119 (10.7%), mobile teeth in 106 (9.5%), and mandibular fractures in 65 (5.8%). Tooth avulsion was observed in 60 patients (5.4%) while fewer cases presented with lockjaw (n=28; 2.5%) and maxillary fractures (n=12; 1.1%).

Conclusion

A high incidence of oro-maxillofacial emergencies, including traumatic injuries & non-traumatic pathologies reported to the emergency department of a tertiary care hospital in six months. This study highlights the need for the promotion of oral health through public awareness to reduce the incidence of non-traumatic oral pathologies and more stringent regulations to mitigate traumatic oro-maxillofacial injuries.

## Introduction

According to a report released by the National Institution for Transforming India (NITI) Aayog, emergency and injury cases annually account for 9-13% of all patients presenting to a tertiary healthcare facility (both government and private) [1]. This report is based on a study conducted by the Department of Emergency Medicine at All India Institute of Medical Sciences (AIIMS), New Delhi. A US-based study in 2018 reported that out of a total of 143 million visits to the Emergency Department (ED), two million were dental-related (1.4%) [2].

The oro-maxillofacial emergencies may originate from intraoral or extraoral hard and soft tissues involving the teeth and surrounding periodontium or mucosa, as well as related maxillo-mandibular spaces. The American Association of Oral and Maxillofacial Surgeons (AAOMS) and the American Dental Association (ADA) have listed the emergency conditions of the orofacial region or dental emergencies that include the jaw and alveolar bone fractures, avulsed or displaced teeth, fractured teeth with pulp exposures, acute alveolar abscess, upper airway impairment, oral mucosal lacerations, acute dental pain and infection, and uncontrolled bleeding [3,4].

A study conducted in the UK reported a total of 211 dental emergencies, comprising 156 infection-related cases, 42 trauma-related cases, and 12 cases of post-operative complications. The reasons for dental emergencies included tooth and gum pain due to caries, periodontitis, bleeding, erupting tooth, pericoronitis, and trauma [5]. Bae et al. reported that dental trauma, dental infection, oral bleeding, and temporomandibular joint disorders were common conditions observed in the emergency room [6]. An online survey conducted among dental practitioners in India revealed that the common dental emergencies reported were tooth pain (100%), swelling (91.7%), decayed tooth (81.3%), and gum pain (78.6%) [7].

Guru Teg Bahadur Hospital (GTBH) is one of the largest healthcare centres in the National Capital Region, Delhi, India. It provides healthcare facilities not only to residents but also to patients from the adjoining areas of other states. An average of 4000 patients report daily to the out-patient department (OPD) of our hospital. The hospital is equipped with round-the-clock emergency services in various clinical specialties. About 25,000 patients visit the ED of GTBH annually. The enormous patient inflow to the ED of our institute prompted us to assess the number, nature, and severity of the emergency cases related to the oral and maxillofacial region. This would also help us identify the risk factors leading to such emergencies. Moreover, the insights gained would help in taking constructive steps to upgrade the facilities in terms of infrastructure and manpower to cater to this densely populated region. The current literature lacks sufficient in-depth analysis regarding the prevalence and types of emergencies related to the oro-maxillofacial region. Therefore, the primary objective of this retrospective study was to investigate the incidence and characteristics of oro-maxillofacial emergency visits to the ED of the hospital for six months. The secondary objectives were to classify the types, anatomical distribution and etiology of oro-maxillofacial emergencies. This will facilitate the establishment of clinical and research priorities for providing effective treatment and quality care for individuals with oro-maxillofacial emergencies.

## Materials and methods

Study design

This study was retrospective, observational, hospital-based, and conducted at the emergency department (ED)/casualty of a tertiary care teaching hospital. The primary objective was to evaluate and classify the range of emergencies related to oro-dental and maxillofacial conditions that presented to the ED of our institution over a defined period. The study aimed to provide a descriptive account of the demographic and clinical presentation of such cases, while also categorizing them based on the chief complaint, anatomical involvement, and etiology. The time frame selected for this study spanned six consecutive months, from January 1, 2024, to June 30, 2024. The sample size for this study was not determined, as it was an observational study with a fixed time frame.

Patient population

Our institute serves a diverse patient population and functions as a major referral center for the region, allowing for a wide range of acute orofacial conditions to be documented. The study population specifically included patients who presented directly to the ED. All patients, irrespective of age or gender, who presented to the ED with primary complaints related to the oro-dental or maxillofacial region during the study period were considered eligible for inclusion. This included clinical conditions such as traumatic injuries (including soft tissue and skeletal injuries), acute infections, severe dental pain, uncontrolled bleeding, mandibular dislocations, and other urgent conditions requiring immediate dental or surgical intervention.

The patients reporting for routine outpatient dental services or emergency cases returning for follow-up visits in the OPD or repeat visits with the same concern to the ED were explicitly excluded from the analysis to maintain the focus on emergency presentations only. All the patients reporting to the ED are assessed for the Glasgow Coma scale, and the patients with scores in moderate or severe categories were not included. Only the patients responsive to commands i.e., with Glasgow Coma Scale (GCS) score of less than 13 were included in the study. Patients with incomplete documentation or missing essential clinical information in their ED records were also excluded.

Data collection

The data collection was carried out in a systematic and structured manner by reviewing patient records maintained in the hospital’s electronic and physical emergency documentation system. These records were accessed through the hospital’s internal records under administrative permission.

Information extracted from the records included the patient’s age, gender, presenting chief complaint, underlying etiology, any diagnostic investigations conducted (e.g., radiographs, CT scans, blood tests), and details of immediate management or interventions provided in the emergency setting. Each patient’s case was reviewed thoroughly to ensure that the primary complaint was appropriately classified. Few cases presented with multiple complaints and were categorized accordingly.

To ensure data privacy and confidentiality, all the patient-identifying information, such as names, contact details, and case registration numbers (CR numbers), was excluded from the dataset. A new dataset was created using Microsoft Excel (Microsoft Corp., Redmond, WA, US), wherein each patient was assigned a unique case identifier to enable systematic analysis without compromising confidentiality.

To ensure accuracy and completeness, the data was independently reviewed by two trained investigators who independently scanned the same set of records and then cross-verified the entries. Any discrepancies or missing entries were resolved by jointly revisiting the source records. This double-checking process helped minimize human error and ensured the robustness of the final dataset.

Data categorization according to etiology

The etiology of each case was also recorded and broadly classified into traumatic, infective, inflammatory, iatrogenic, neoplastic, or idiopathic categories, wherever such classification was applicable based on available clinical records. The study did not require active patient consent as it was retrospective and involved the use of previously recorded clinical data without any direct patient interaction or intervention. All data were anonymized and handled with strict adherence to ethical standards for patient confidentiality. 

Statistical analysis

All analysis was performed on a computer using IBM SPSS Statistics for Windows, Version 23 (Released 2015; IBM Corp., Armonk, New York, United States). Multivariate logistic regression was used to correlate the risk factors for maxillofacial soft tissue trauma. The Pearson Chi-square value and odds ratio (OR) were calculated to assess this correlation. The risk factor for fracture affecting a particular mandibular site was evaluated using the Pearson Chi-square test.

## Results

The examination of records yielded 1,117 patients with oro-maxillofacial emergencies. The various presenting complaints related to the oro-maxillofacial region in the ED included pain in a carious tooth, wisdom tooth, avulsed tooth due to trauma, tooth fracture, mobile tooth, soft tissue laceration, and maxillary or mandibular fracture. Radiographic examinations were used to confirm the site of fracture. Mandibular fractures were classified using Dingman and Anvtig classification, while midfacial fractures were categorized according to Le-Fort classification [8,9].

Data normality was assessed using the Kolmogorov-Smirnov test and found to be non-normal; therefore, non-parametric tests were applied. Categorical variables are expressed as the number of cases with percentages where appropriate. Associations between variables were analyzed using the Pearson Chi-square test; p<0.05 was considered statistically significant (p<0.01 highly significant). Odds ratios (OR) with 95% confidence intervals (CI) were calculated where applicable.

Epidemiological characteristics

**Figure 1:**
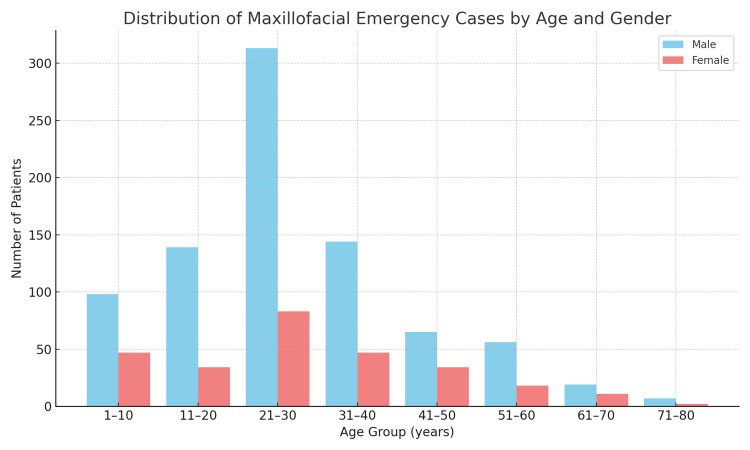
Age and gender distribution of the patients with oro-maxillofacial emergencies

The mean age of patients with oro-maxillofacial emergencies was 28.75±15.43 years, ranging from one to 84 years. Figure 1 presents the age and gender distribution of these patients.

The majority of the patients were in the 21-30 years age group (n=396, 35.5%), followed by the 31-40 years age group (n=191, 17.1%). A male preponderance was observed (n=841, 75.3%), with a male to female ratio of 3.04:1 (χ²=17.57, p=0.025) (Figure 1).

The data were compiled based on the type of presenting complaint, its site, and the etiology of the complaint (Table 1).

**Table 1:** Oro-maxillofacial emergencies reporting to the emergency department RTA: Road traffic accidents; Data are expressed as n (%); Chi-square test was used for analysis.

Type of emergency	Tissue	Maxillofacial emergency	Total	(%)	RTA	Fall	Assault	Firearm	Sports	Animal	Fire	Train	Natural disaster	Other
Non-traumatic dental emergencies	Teeth	Pain wisdom	40	3.6										
		Pain carious	168	15										
Traumatic dental emergencies	Soft tissue	Injury	506	45.3	187 (37)	150 (29.6)	148(29.2)	0	8 (1.6)	0	0	0	1 (0.2)	12
		Facial swelling	119	10.7	22 (18.5)	27 (22.7)	61 (51.3)	0	5 (4.2)	0	0	0	0	4
	Hard tissue injury bone	Maxillary fracture	12	1.1	6 (50)	0	5 (41.7)	1 (8.3)	0	0	0	0	0	-
		Mandibular fracture	65	5.8	30 (46.2)	14 (21.5)	15 (23.1)	1(1.5)	1(1.5)	0	0	0	0	-
	Hard tissue injury dentoalveolar	Avulsion	60	5.4	12 (20)	40 (58.3)	7 (11.7)	0	1 (1.7)	0	0	0	0	0
		Tooth fracture	136	12.2	42 (30.9)	50 (36.8)	36 (26.5)	0	1 (0.7)	0	0	0	0	7
		Mobile tooth	106	9.5	26 (24.5)	40 (37.7)	29 (27.4)	0	3 (2.8)	1 (0.9)	0	0	1 (0.9)	6
Others		Lock jaw	28	2.5	1 (3.6)	5 (17.9)	3 (10.7)	0	0	0	0	0	0	19

Types of oro-maxillofacial emergencies

Non-traumatic emergencies included pain in wisdom teeth or carious teeth, i.e., oro-maxillofacial emergencies, chiefly associated with the dental causes. A total of 208 patients reported with non-traumatic dental emergencies, including 168 patients (102 male and 66 female patients) who experienced pain in carious teeth and 40 patients (22 male and 18 female patients) with pain in wisdom teeth. The majority of these non-traumatic cases were in the age groups of 21-30 and 31-40 years.

Traumatic emergencies comprised soft tissue or hard tissue injuries involving the oro-maxillofacial region. Out of a total of 1,117 such emergencies, 506 (45.2%) patients had soft tissue injuries, 136(12.2%) had tooth fractures, 119 (10.7%) had facial swelling, 106 (9.5%) had mobile teeth, and 65(5.8%) patients had mandibular fractures. Avulsion of the tooth was reported in 60 (5.4%), while a lower percentage of cases presented with lock jaw (n=28, 2.5%) and maxillary fractures (n=12, 1.1%).

The soft tissue injuries ranged from abrasions to deep lacerations, including insults to the oral mucosa or facial skin of the maxillofacial region. The tooth fracture mainly involved the anteriors, especially in the upper arch, frequently affecting the maxillary central incisors. 

Risk factors/etiology of traumatic emergencies

Soft Tissue Injuries

For soft tissue injuries, the most common cause was road traffic accidents (RTA) (n=187), followed by falls (n=150) and assaults (n=148). The main cause of facial swelling was assault (n=61), followed by falls (n=27) and RTAs (n=22). Table 2 shows the correlation of each of these factors reportedly causing soft tissue injuries.

**Table 2:** Risk factors/ etiology of soft tissue injuries RTA: Road traffic accidents; *p<0.05 considered statistically significant. Pearson Chi-square test was used to assess association. Odds ratio (OR) with 95% confidence interval (CI) reported.

Soft tissue injury	Pearson Chi-square	P value	Odds Ratio (OR)	95% Confidence Interval lower	95% Confidence Interval upper
RTA	72.07	0.000*	3.35	2.51	4.46
Fall	14.07	0.000*	1.68	1.28	2.224
Assault	11.06	0.001*	1.59	1.2	2.09
Fire arm	0.829	1	-	-	-
Sports	0.145	0.802	1.21	0.451	3.24
Animal	0.829	0.363	-	-	-

Hard Tissue Injuries

Bone: Out of all the traumatic oro-maxillofacial emergencies, 77 cases (8.48%) presented with maxillofacial fractures. The most common site of fracture was the mandible (n=65, 5.8%), followed by the maxilla (1.1%).

Maxillary fractures: Amongst the 12 maxillary fractures, the main cause was RTA in six cases, followed by assault in five cases.

Mandibular fractures: 65 cases were reported with mandibular fractures. The main cause for mandibular fracture was RTA (n=30), followed by assault (n=15) and fall (n=14).

**Figure 2:**
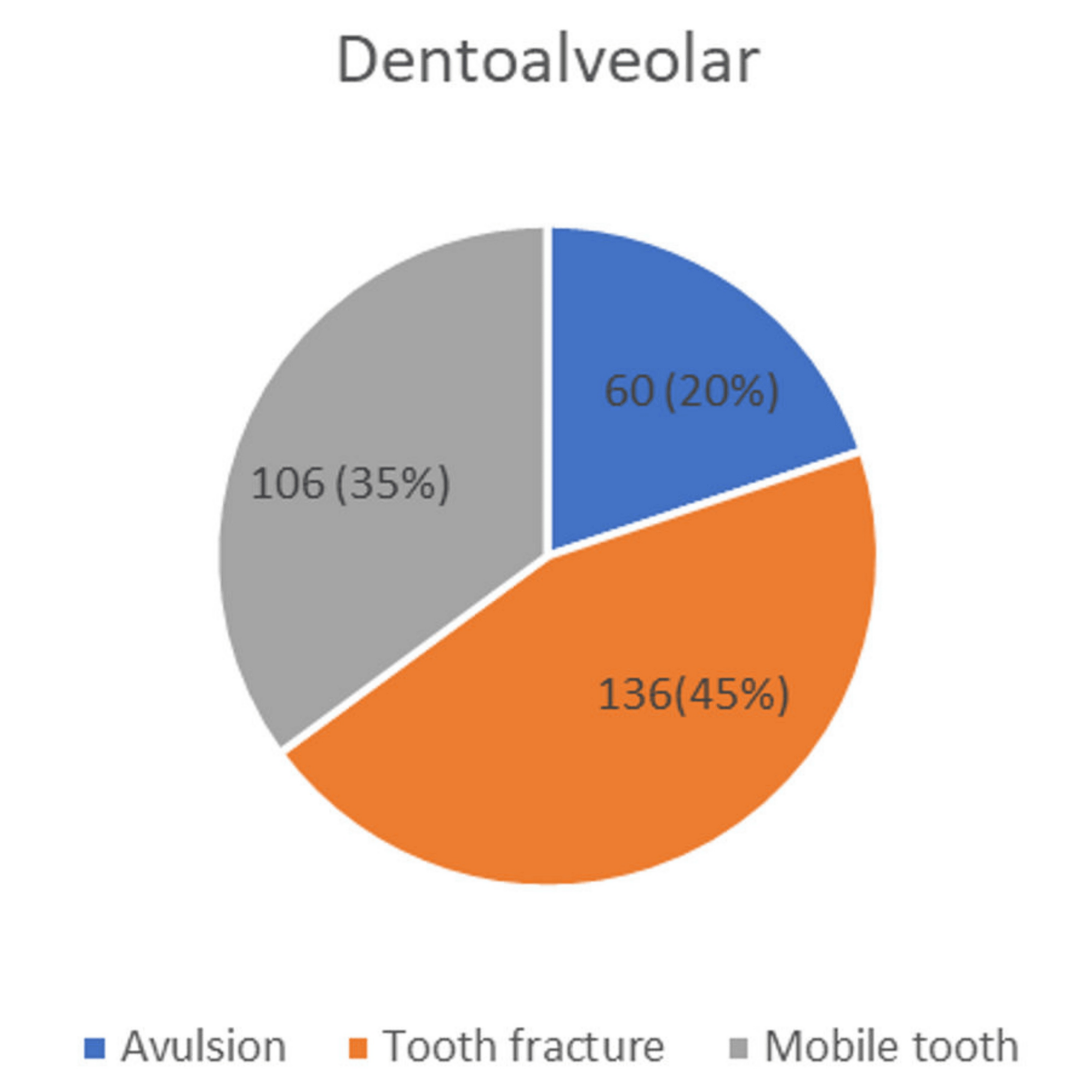
Distribution of dentoalveolar injuries

Dentoalveolar: Dentoalveolar injuries, including tooth fractures, were observed in 136 (45%) patients, mobile tooth in 106 (35%), and avulsion in 60 (20%) patients (Figure 2).

Tooth fractures were primarily caused by falls (n=50), followed by RTAs (n=42) and assaults (n=36). The etiology of avulsion was mainly falls (n=40). However, the causative factors for this emergency were not reported in the dental notes. The cause of tooth mobility was nearly equal for RTA and assault in 29 and 26 cases, respectively, and was not recorded for six cases. Thus, the etiology in six cases that reported with mobility was not mentioned in the records.

Other emergencies: Out of the 28 patients who reported with the problem of lock jaw, fall (n=5), assault (n=3), and RTA (n=1) were amongst the definitive causative factors as per the records. However, for the maximum number of patients (n=19), no definitive cause was registered against this complaint.

Besides trauma being the most probable cause for tooth fracture and facial swelling, the definitive cause could not be determined in seven cases with fractured teeth and four cases with facial swelling.

Maxillofacial traumatic injuries: site and age predilection

As evident from Table 3, the predominant site for mandibular fracture was the para-symphysis region (n=46), followed by the symphysis (n=11), condylar (n=7), and angular (n=3) regions.

**Table 3:** Site distribution of mandibular fractures vs etiology Data are presented as number of cases. Pearson Chi-square test was used to assess the association between fracture site and etiology. p<0.05 considered statistically significant (p<0.01 highly significant).

Mandibular fracture	Total	RTA	Fall	Assault	Firearm	Sports
Symphysis	n=11	n=6	n=2	n=2	0	n=1
		X^2 ^=5.22	X^2 ^=0.23	X^2 ^=0.21		X^2 ^=4.61
		P=0.03	P=1.000	P=1.000		P=0.14
Para-symphysis	n=46	n=22	n=11	n=10	n=1	n=1
		X^2 ^=12.61	X^2 ^=0.024	X^2 ^=0.224	X^2 ^=22.78	X^2 ^=0.168
		P=0.01	P=0.03	P= 0.730	P=0.042	P=0.50
Condylar	n=7	n=2	n=1	n=3	0	0
		X^2 ^= 0.000	X^2 ^=0.61	X^2 ^=0.781		
		P= 1.000	P=0.688	P=0.409		
Angular	n=3	n=2	0	n=1	0	0
		X^2 ^= 2.89				
		P=0.155				

According to the age group, the proportion of patients with maxillofacial traumatic injuries was higher in the 21-30-year age group (n=318), followed by the 31-40-year age group (n=150) (Table 4).

**Table 4:** Age distribution and cause of injury RTA: Road traffic accidents; Values are expressed as number of cases; Pearson Chi-square test was used.

Age group (years)	RTA	Fall	Assault
1–10	18	99	11
11–20	42	46	45
21–30	113	72	133
31–40	60	38	52
41–50	30	15	29
51–60	22	16	11
61–70	9	5	7
71–80	1	3	0
81–90	0	0	0
Total	295	294	288

## Discussion

The emergency services are an important criterion that defines the proficiency of tertiary care hospitals in catering to the needs of society. The OPD services in such hospitals are generally deemed advanced, and thus, are considered to be at the apex in the hierarchy of referral centers. However, the status of the emergency services, especially concerning dental emergencies as well as maxillofacial trauma, has seldom been assessed. Dental emergencies have been broadly categorized based on etiology as originating from trauma, infections, and post-procedural complications [10]. Though the oro-maxillofacial emergencies arising from trauma have frequently been examined in the literature, the studies reporting the combined load of all types of dental/ maxillofacial emergency cases encountered in a healthcare setup are scarce. Hence, this study was undertaken to evaluate the oro-maxillofacial emergency cases comprehensively, including the patient load, nature, type, distribution, and risk factors for such emergencies.

It is essential to critically examine the records of patients reporting with emergencies like oro-maxillofacial pathology or trauma, both to analyze the causes behind such emergencies, as well as to maintain adequate facilities for their successful management. This would also help in restricting the hard and soft tissue damage and minimize the complexity of secondary or more elaborate procedures for rehabilitation. Such epidemiological studies form the basis of national healthcare policies and provide guidelines for resource allocation for management of healthcare emergencies [11].

The analysis of the data collected from the ED of our hospital revealed a total of 1,117 patients who visited for dental emergency care over six months. This number is nearly equal to the annual emergency cases reported in similar hospitals like Seoul National University, Bundang Hospital (SNUBH) emergency center in Gyeonggi-do, Korea (1,425 patients from January to December 2009) [6]. Royal Hobart Hospital, Australia, reported that 444 patients had presented to the ED with dental complaints in 2012 (0.91% of all ED presentations) [12]. In the current study, the incidence of dental emergencies in male patients was nearly three times that seen in female patients, and the majority of cases were in the age group of 21-30 years (37.2% & 30.02% for male and female patients, respectively).

The non-traumatic emergencies related to the orofacial region are a sequelae of dental infections. If untreated, these dental infections may progress from pulpitis to pulp necrosis and periapical abscess. The periodontal or pericoronal tissue in the case of unerupted teeth, especially third molars, may also be infected or inflamed directly or through the infectious spread from dental tissues [13,14]. In a study by Hutomo et al., out of 774 dental patients visiting the emergency room at the Airlangga University Dental Hospital, 366 patients (47.7%) had infections recorded as the main reason for the visit, while 166 (21.8%) had caries, 92 (12.1%) had trauma, 45 (4.9%) had bleeding, 11 (1.4%) had temporomandibular disorders, and 92 (12.1%) had various other complaints [15]. This is in contrast to our findings, where only 18.6% patients reported with dental pain of non-traumatic origin. However, since these types of emergencies can readily be minimized by regular check-ups and dental health awareness, even this low percentage of cases is a matter of concern. Effective preventive and public dental health education measures are essential to reduce the burden and incidence of oro-maxillofacial emergencies of dental origin [16].

The percentage of traumatic insults to the oro-maxillofacial tissues was found to be alarming (81.4%) in our study. These injuries involved the soft tissues alone in the majority of patients, including intra-oral and extraoral abrasions or lacerations affecting the skin or the mucosa. Since the face is a region of extreme aesthetic and functional significance, such injuries need urgent attention as they may affect the psychosocial status of the individual in severe cases due to scarring and facial disfigurement [17]. These injuries are further complicated by the fact soft tissue in this region numerous vital structures like vessels, ducts, nerves, and muscles. The possibility of foreign debris incorporation and hematoma are other threats that make such injuries critical [18]. A study by Khan et al. reported a high frequency of multiple lacerations (38%) in the maxillofacial region [19].

Facial swelling generally accompanies numerous pathological and traumatic causes. Untreated dental caries may progress to apical swelling, abscess, sinus, or in extreme cases, to cellulitis or infection spreading along the fascial planes with resultant swelling evident on the face [20,21]. In the current study, only traumatic facial swelling without underlying bony or dentoalveolar damage was recorded as a separate clinical finding. The finding of soft tissue injury in 10% of trauma cases reporting to the ED is similar to that reported by Ketlow et al. [22]. Since nearly half of the cases presented with soft tissue injuries, the etiological factors associated with these were segregated. Such injuries occur due to various reasons like road traffic accidents (RTAs), physical assaults, accidental falls, industrial mishaps, sports injuries, and firearm injuries [23-25]. In the current study, RTAs emerged as the major cause of such injuries.

Hard tissue injuries to the tooth, like fractures, mobility, and avulsion, together accounted for 27.1% of cases. While the most probable cause of tooth fracture and avulsion would have been a traumatic episode, the mobility of the tooth may have had a periodontal cause or component to it. The injuries to teeth ranged from enamel grazing, enamel-dentin fracture, to pulpal exposure. Bourguignon et al. have summarized the various types of tooth injuries along with guidelines for their management [26]. The anterior teeth are more vulnerable to fracture owing to their location. Their forward placement or proclination further adds to this risk [27]. 

Avulsion of teeth has been reported to be 0.5%-16% of all dental injuries in permanent teeth [28]. This is one of the most serious dental injuries, and timely intervention to reimplant the tooth may give a new lease of life to the avulsed tooth. However, many times it may not be possible due to the lack of proper emergency management, loss of tooth, or other technical issues. The prognosis of a reimplanted tooth in the long term may not be favorable even after appropriate emergency care [28].

Trauma centers worldwide record oro-maxillofacial fractures as one of the most common sequelae of traumatic injuries [11]. In the current study, nearly 6% of dental patients examined in the ED presented with mandibular fractures, which is 65% of the total maxillofacial fractures recorded. In a review by Adeleke, the mandible was the most frequently involved bone in the maxillofacial injuries [29]. While the parasymphysis was seen as the most common site of mandibular fracture in the current study (Table 3), similar to Adeleke's observations [29], the angle of the mandible was the least commonly involved site. Avery et al. have given a diagrammatic outline of the site susceptibility in mandibular fractures based on three-dimensional CT evaluation of patients with facial injuries [30]. 

Since the soft tissue injuries accounted for the maximum number of cases, the etiology of such injuries was analyzed in detail (Table 2). RTAs were the single most common cause of these injuries. The various causes of maxillofacial injuries as seen in our study are similar to those reported by Singaram et al. [31]. The age distribution of the patients presenting with oro-maxillofacial trauma revealed that the maximum number of them fell in the age range of 21-40 years, irrespective of the etiology of the injury, which is consistent with Singaram et al. [31]. The possible explanation for this observation lies in the fact that this age group is the most active population of the society, with maximum exposure to risk situations. This results in higher incidences of RTA, fall, assault, etc., causing oro-maxillofacial trauma in this age group.

The incidence of maxillary fractures was found to be relatively low in the current study. Rosello et al. reported that the maxilla is the second or third most common facial bone to be fractured in facial trauma, as per different studies [32]. This region is often affected by complex fractures involving multiple midfacial bones that increase the risk and severity of associated complications. The number of cases presenting with lockjaw is low in terms of percentage (2.5%), but still noteworthy. Llgunas et al. reported an annual incidence rate of 1.1% (women) and 0.5% (men) for jaw catching/locking in the Swedish population [33]. The lock jaw may result from trismus, i.e., sustained tetanic spasms of the muscles of mastication, or due to temporomandibular dysfunction consequent to trauma or degenerative joint changes [33].

The limitation of this study was that only records from the ED were examined. Many of the oro-maxillofacial emergency cases may directly report to the OPD, especially those of dental origin (infectious), and hence have not been included in the analysis.

## Conclusions

The present study highlights the considerable burden of oro-maxillofacial emergencies encountered in the emergency department of a tertiary care hospital. A notable proportion of these emergencies is attributable to the diseases/conditions of dental origin. This highlights the importance of strengthening preventive oral health programs and the need for dental health education in the society. Public dental awareness campaigns, timely access to primary dental care, and early interventions can curtail the progression of various dental diseases. This would significantly reduce the likelihood of minor dental ailments from developing into dental emergencies.

Moreover, the great proportion of dental emergencies that result from traumatic incidents like RTAs and interpersonal violence emphasize the need for robust public health measures, including stricter enforcement of traffic regulations, promotion of road safety practices, and community initiatives aimed at reducing violence. Collectively, these findings underscore the dual need for preventive strategies in both dental and trauma-related domains to reduce the overall burden of oro-maxillofacial emergencies on healthcare services.
